# Determining the Dielectric Tensor of Microtextured Organic Thin Films by Imaging Mueller Matrix Ellipsometry

**DOI:** 10.1021/acs.jpclett.1c00317

**Published:** 2021-03-19

**Authors:** Sebastian Funke, Matthias Duwe, Frank Balzer, Peter H. Thiesen, Kurt Hingerl, Manuela Schiek

**Affiliations:** † Accurion GmbH, Stresemannstr. 30, D-37079 Göttingen, Germany; ‡ Centre for Photonics Engineering, University of Southern Denmark, Alsion 2, DK-6400 Sønderborg, Denmark; § Center for Surface- and Nanoanalytics, Johannes Kepler University, Altenbergerstr. 69, A-4040 Linz, Austria; ∥ Institute of Physics, University of Oldenburg, Carl-von-Ossietzky-Str. 9-11, D-26129 Oldenburg, Germany

## Abstract

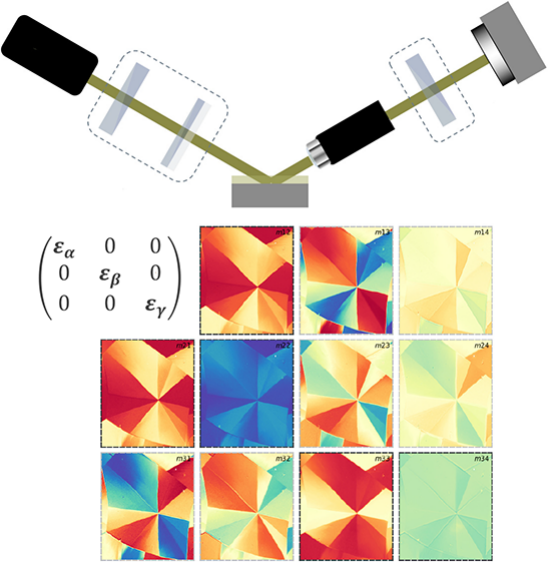

Polycrystalline textured thin films with distinct pleochroism and birefringence comprising oriented rotational domains of the orthorhombic polymorph of an anilino squaraine with isobutyl side chains (SQIB) are analyzed by imaging Mueller matrix ellipsometry to obtain the biaxial dielectric tensor. Simultaneous fitting of transmission and oblique incidence reflection Mueller matrix scans combined with the spatial resolution of an optical microscope allows to accurately determine the full biaxial dielectric tensor from a single crystallographic sample orientation. Oscillator dispersion relations model well the dielectric tensor components. Strong intermolecular interactions cause the real permittivity for all three directions to become strongly negative near the excitonic resonances, which is appealing for nanophotonic applications.

Semiconductor thin films are technologically relevant for optoelectronics and photonics. Typically, they are micro- or nanotextured and anisotropic in their structural and resulting optoelectronic properties.^1−4^ Here, it is crucial for fundamental and applied research to have a quantitative understanding of light–matter interactions. These are basically described by the complex dielectric function, which is a tensor quantity. Knowledge of the full dielectric tensor allows to calculate light propagation and attenuation in arbitrary lattice directions, which is of universal practical relevance. The small-scaled texture of crystalline domains in polycrystalline materials impedes full acquisition of the dielectric tensor by global ellipsometric approaches.^5^ Imaging Mueller matrix ellipsometry^6−9^ combines the power of variable angle spectroscopic ellipsometry^10−12^ and optical microscopy mapping to obtain the complete complex dielectric tensor of microtextured biaxial anisotropic thin film samples from even a single crystallographic orientation.

In this study we investigate polycrystalline organic thin films of a model anilino squaraine with isobutyl side chains (SQIB). The material has been considered for photovoltaic applications^13−15^ and implemented in studies on fundamental light–matter interactions.^16^ Here, the samples consist of birefringent and pleochroic rotational platelet-like SQIB domains crystallized in its *Pbcn* orthorhombic polymorph, oriented with the (110) plane parallel to the substrate. The unit cell parameters are *a* = 15.0453 Å, *b* = 18.2202 Å, *c* = 10.7973 Å, α = β = γ = 90°, and *Z* = 4. The crystallographic data file is available from the Cambridge Structural Database under the code CCDC 1567104 for full structural information.^17^ A typical polarized reflection microscopy image of a SQIB platelet sample prepared by spin-coating on a glass substrate with subsequent thermal annealing at 180 °C can be seen in Figure 1a. The platelets have variable rotational in-plane orientation, which can be deduced from the golden-to-dark contrast. This contrast is sharp at platelet boundaries, but sometimes there is also a gradual flow of contrast noticeable within a platelet subdomain. The average domain size depends on the processing parameters (see Supporting Information Figure S1 and associated text). Atomic force microscopy (AFM) reveals a certain undulate surface roughness (see Figure S2). The domain size ranges from few tens to several hundred micrometers, making them well suited for spatially resolved optical spectroscopic and ellipsometric imaging investigations.

**Figure 1:**
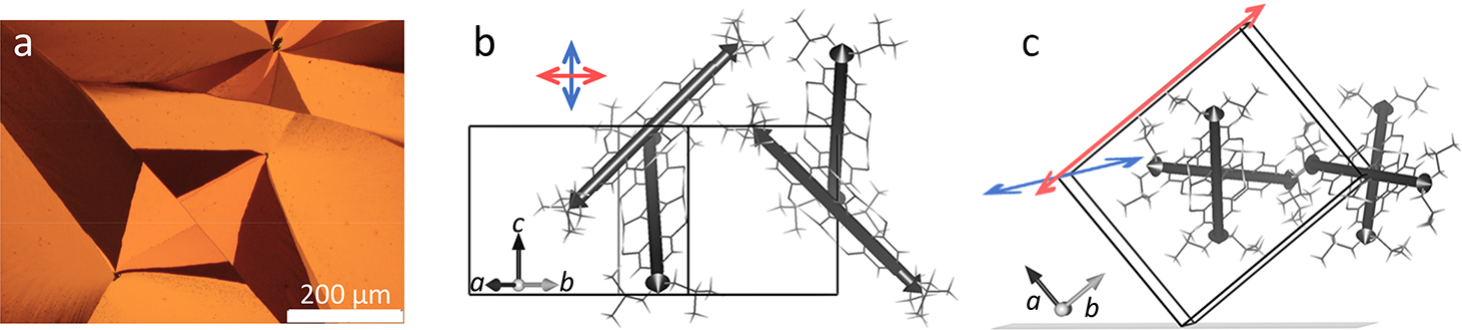
(a) Reflection microscopy image between crossed polarizers (Olympus BX41) of orthorhombic SQIB platelets. (b) Projection of the *Pbcn* single crystal structure with view onto the (110) plane, which is the preferred adopted orientation of the platelets. All four molecules of the unit cell are sketched. Their main transition dipole moment (TDM) is parallel to the long molecular axis, which is indicated by a black arrow. The cross marks the projected directions of LDC (red, horizontal arrow) being along the projection of the *a*- and *b*-axis and UDC (blue, vertical arrow) being along the crystallographic *c*-axis. (c) Side view almost along the *c*-axis (direction of molecular π-stacking) lying within the substrate plane illustrates how the unit cell stands on its *c*-axis edge within the SQIB platelets. The blue and red arrows depict the direction of UDC (parallel to *c*-axis) and LDC (parallel to *b*-axis), respectively. They have been visualized by using VESTA^18^ as the geometric vector sum for repulsive (subtraction, UDC) and attractive (addition, LDC) alignment of all four TDMs indicated by black arrows per unit cell. The lengths of the red and blue arrows are arbitrary for illustration purposes of the directions of UDC and LDC only.

In previous studies^17,19^ we have investigated the local excitonic properties of such platelets showing a pronounced Davydov splitting based on the existence of four nonequivalent molecules within the unit cell.^20^ For completeness, local polarized absorbance spectra recorded in normal incidence onto the (110) plane are shown in Figure S3. The view onto the (110) plane of the single-crystal structure is sketched in Figure 1b, which is the perspective of the normal-incidence spectromicroscopic measurements. This indicates that the upper Davydov component (UDC) is polarized along the crystallographic *c*-axis (molecular stacking direction), while the lower Davydov component (LDC) is polarized along the projection of the *a*- and *b*-axes. The red and blue arrows in Figure 1b indicate the polarization directions of LDC and UDC, respectively. These directions are in coincidence with the result from a graphical vector addition of the projected transition dipole moments (TDMs) along the long molecular axis of all four translationally invariant molecules per unit cell. For a 3-dimensional perspective a side view onto the crystallographic unit cell standing on the *c*-axis edge, almost along the [001] direction, is sketched in Figure 1c. Geometric vector addition/subtraction (performed with VESTA^18^) of the TDMs of all four molecules in the unit cell reveals that UDC (blue arrow) is polarized parallel to the *c*-axis while LDC (red arrow) is parallel to the *b*-axis. For the latter, only its projection onto the (110) plane could be seen in the previous normal-incidence spectromicroscopic measurements.^17,19^

For an orientation-independent, quantitative understanding of the orthorhombic SQIB polymorph’s optical properties, knowledge of the dielectric tensor ε̃ is required. In orthorhombic crystals, the principal axes of both the real and imaginary part of the dielectric tensor coincide.^21^ The dielectric tensor relates to the complex refractive indices along the axes of the index ellipsoid (or optical indicatrix) via
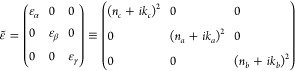
1with Re(ε_α,β,γ_) = *n*_α,β,γ_^2^ – *k*_α,β,γ_^2^ and Im(ε_α,β,γ_) = 2*n*_α,β,γ_*k*_α,β,γ_. The principal axes of ε̃ also coincide with the crystallographic axes.^22^

For an anisotropic arrangement of polycrystalline thin film samples, the orientation of the dielectric index ellipsoid α, β, and γ with respect to the Cartesian laboratory coordinate system *x*, *y*, *z* is described by the Euler angles ϕ, θ, and ψ (eq S1 of the Supporting Information). The angle ϕ describes counterclockwise azimuthal rotation around the *z*-axis, θ describes tilting of the *z*-axis, and ψ is another rotation around the tilted *z*′-axis. In case of the SQIB platelets the (110) out-of-plane orientation is fixed, but the in-plane orientation of platelet domains is variable. This means that ϕ must be a fit parameter freely variable for each domain. The ϕ orientation is given by the crystallographic *c*-axis orientation and is assigned as the indicatrix axis α during the fitting procedure, meaning ε_α_ = ε_*c*_. The Euler angle θ can be set to a fixed angle according to the adopted out-of-plane orientation. In the present case, the crystallographic unit cell stands on the edge given by the *c*-axis as illustrated in Figure 1c. If θ denotes the tilt angle of the crystallographic *b*-axis with respect to the surface normal, then its value can be preset to 50.5° based on the unit cell data. The *b*-axis is assigned as indicatrix axis γ during the fitting procedure; thus ε_γ_ = ε_*b*_. This implies that the tensor element along β describes the polarizability along the *a*-axis, i.e., ε_β_ = ε_*a*_. The Euler angle ψ can be kept at 0° and remains unconsidered within the complete fitting routine.

The biaxial anisotropy of the SQIB sample desires Mueller matrix ellipsometry for determination of the full dielectric tensor. However, for absence of depolarization effects the Jones matrix is also sufficient. But in any case, the microsized grain texture demands spatial resolution of the recordings. To accomplish this task, we used a NanoFilm_EP4 imaging Mueller matrix ellipsometry system (Accurion GmbH, Göttingen) as sketched in Figure 2a. Details on the instrument and the measurement procedure can be found in the Supporting Information. Briefly, the instrument has a polarizer–compensator–sample–analyzer (PCSA) configuration with a rotating compensator allowing to record 11 normalized out of 16 Mueller matrix elements. A 10× long working distance objective before the analyzer and a CCD camera for signal detection yield lateral resolution down to 2 μm without the need for a tightly focused probing beam. Incoherent reflections from the backside of the glass substrate are suppressed by knife edge illumination^8^ (see Figure S4). Each acquired pixel contains information on spatial *x*–*y* and spectral wavelength position for the 11 measured Mueller matrix elements. Figure 2b shows the *m*13 element of a normal incidence transmission Mueller matrix scan probing at 596 nm on the sample area of choice recorded with 10× magnification. The imaged SQIB platelet consists of numerous subdomains, from which 14 triangular regions of interest (ROIs) were selected for further analysis. All ROIs have been fitted in the following procedure, both independently and collectively.

**Figure 2:**
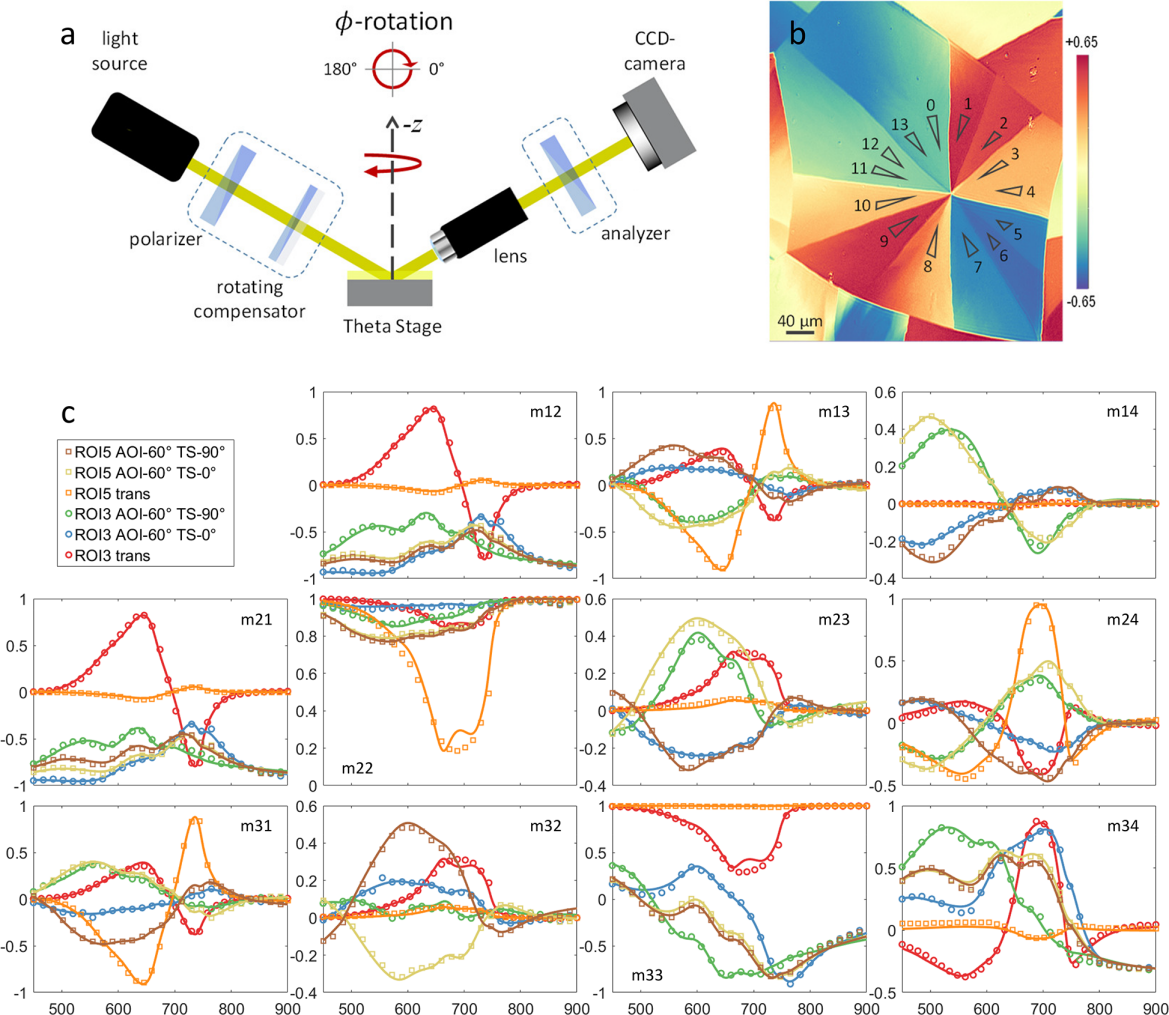
(a) Sketch of the NanoFilm_EP4 imaging Mueller matrix ellipsometer with PCSA configuration. (b) The snapshot from a spatially resolved spectroscopic Mueller matrix measurement in reflection (AOI 50°) through a 10× objective (Nikon, NA 0.21) shows the *m*13 element at 598 nm. The normalized value is color-coded; scale bar from −0.65 to +0.65 rad. Fourteen subdomains of the SQIB platelet are marked by triangular ROIs for data analysis. In (c) the complete spectral courses of the measured Mueller matrices of ROIs 3 and 5 are plotted: normal incidence transmission (trans) and reflection Mueller matrix data at AOI 60° for two Theta Stage (TS) positions at 0° and 90° azimuthal rotation are shown. The circles (ROI 3) and squares (ROI 5) display the measured data with only every seventh data point plotted for clarity, while the solid lines show the fit results. *Y*-axes: normalized Mueller matrix values. *X*-axes: wavelength in nanometers. The complete measured and fitted spectroscopic Mueller matrix data can be seen in Figures S3 and S4.

As a first step, Theta Scans have been recorded in reflection (angles of incidence (AOIs) 50° and 60°) at three fixed wavelengths: 596, 662, and 710 nm. For each wavelength, the sample stage (Theta Stage) is rotated around the *z*-axis to collect all 11 Mueller matrix elements depending on the azimuthal rotation angle in steps of 15°. The measured and fitted Theta Scan data for ROI 0 are exemplarily plotted in Figure S5 together with an explanation of the fitting routine. From the data sets of all 14 ROIs the layer thickness *d*, the rotational domain orientation ϕ, and the tilt angle θ have been determined for all SQIB subdomains. The resulting values are listed in Table 1.

**Table 1:** Fit Results from the Theta Scans for Each of the 14 ROIs

ROI	*d* (nm)	ϕ (deg)	θ (deg)
0	51.8 ± 0.3	253.90 ± 0.01	49.5 ± 0.5
1	52.2 ± 0.3	296.60 ± 0.02	49.3 ± 0.5
2	52.6 ± 0.4	116.20 ± 0.02	48.0 ± 0.5
3	53.0 ± 0.3	–16.27 ± 0.02	49.5 ± 0.5
4	53.1 ± 0.3	345.17 ± 0.02	49.5 ± 0.5
5	51.9 ± 0.4	43.12 ± 0.02	48.9 ± 0.6
6	51.9 ± 0.3	57.99 ± 0.01	49.0 ± 0.5
7	51.3 ± 0.4	240.18 ± 0.01	48.9 ± 0.5
8	50.0 ± 0.4	278.60 ± 0.02	48.4 ± 0.5
9	52.2 ± 0.3	317.05 ± 0.01	49.7 ± 0.5
10	53.1 ± 0.3	–13.84 ± 0.01	49.9 ± 0.5
11	52.6 ± 0.3	206.59 ± 0.01	49.6 ± 0.5
12	52.5 ± 0.3	207.44 ± 0.01	49.5 ± 0.5
13	51.8 ± 0.3	252.42 ± 0.02	49.1 ± 0.5
all	51.7 ± 0.02		49.34 ± 0.04

a Layer thickness *d*, ϕ in-plane orientation angle of crystallographic *c*-axis, and tilt angle θ of the crystallographic *b*-axis. The ϕ orientation is given as the angle of a pointer starting at “3 o’clock” (0° position) rotating clockwise for positive angle counting; see also Figure 2a. MSE = 10^–4^ for simultaneous fitting (last row “all”).

On average over all ROIs the layer thickness *d* amounts to 52.1 ± 0.8 nm while simultaneous fitting of all ROIs returns *d* = 51.7 ± 0.02 nm. To visualize the variability of *d* and its parameter correlation to the values of the dielectric function’s tensor elements, they are plotted in Figure S6 as determined from the Theta Scans of all ROIs. The tilt angle θ of the crystallographic *b*-axis with on average 49.3 ± 0.4°, or 49.34 ± 0.04° for combined fitting of all ROIs, is in good agreement with the calculated 50.5° based on single crystal data for (110) alignment. However, a minimal systematic deviation from the unit cell parameters is possibly present due to the thin film nature of the samples.^23^

Next, spectroscopic Mueller matrix mapping has been carried out both in reflection (AOIs 50° and 60°, Theta Stage at 0° and 90°) and under normal incidence transmission. Selected measured and fitted spectroscopic scans from ROIs 3 and 5 are shown in Figure 2c. The complete data sets for all ROIs can be found in Figures S7 (transmission data) and Figure S8 (reflection data AOI 60°). Spatial mapping images at fixed wavelength are displayed in Figure S9. The layer thickness *d*, tilt angle θ, and the ϕ orientation were used as determined from the Theta Scans for the following fitting procedure of the spectroscopic Mueller matrix data, as detailed out in the Supporting Information. However, a ϕ-offset was allowed for the transmission data to account for a slight misalignment due to different sample adjustment. The transmission measurement was crucial for parameter decorrelation.^23^ Reflection scans at variable AOIs and Theta Stage positions enabled the determination of the full dielectric tensor.^24^ Owing to the orthorhombic symmetry, this was possible from only a single crystallographic orientation.^25^

Analyzing the spectroscopic data was initialized by a batch fit, which is a model-free best match to the measured data points, to give the dielectric tensor components along the three principal axes α, β, and γ. As a final step, the fit was parametrized with sets of Lorentz and Tauc–Lorentz oscillators to secure Kramers–Kronig consistency and to reduce the number of fit parameters. All fitting parameters are tabulated in Figure S10. However, we do not attempt to assign these oscillators to specific excitonic transitions since the crystal band structure is not known yet.

The real and the imaginary part of the dielectric tensor components are graphed in Figure 3a,b as a final result from the combined ROI fitting for transmission and reflection data. To illustrate the reasonably low experimental spread of the tensor components, the individual fitting results for all ROIs are displayed together with the combined final fit data in Figure S11. The same data but in the representation as complex refractive index, refractive index *n* and extinction coefficient *k*, are given in Figure S12a,b. Compared to inorganic semiconductors, the dielectric function of organic crystals such as SQIB shows to a much smaller amount the influence of band structure and Van Hove singularities^26^ but is dominated by excitonic transitions possibly also including vibronic replicas.^20^ The tensor component ε_α_ (graphed in blue in Figure 3a,b) is congruent with the crystallographic *c*-axis and gives UDC, while LDC is described by ε_γ_ (graphed in red) being along the *b*-axis. Actually, the largest polarizability is along the crystallographic *a*-axis, which is described by ε_β_ (graphed in yellow). The peak values of the imaginary part of LDC and ε_β_ are at 743 nm (1.669 eV) and 730 nm (1.698 eV), respectively, and have very similar full width at half-maxima (FWHM) of 53 ± 1 meV. The UDC is blue-shifted and peaks at 652 nm (1.902 eV) with a certainly larger FWHM of 122 meV. This can be understood from molecular exciton theory saying that UDC behaves like an H-aggregate while LDC like a J-aggregate.^20^ Thus, UDC contains vibronic progressions, which are unresolved here, but appear as spectral broadening. The Davydov splitting as peak-to-peak energy between UDC and LDC calculates to 233 meV from the respective tensor components.

**Figure 3:**
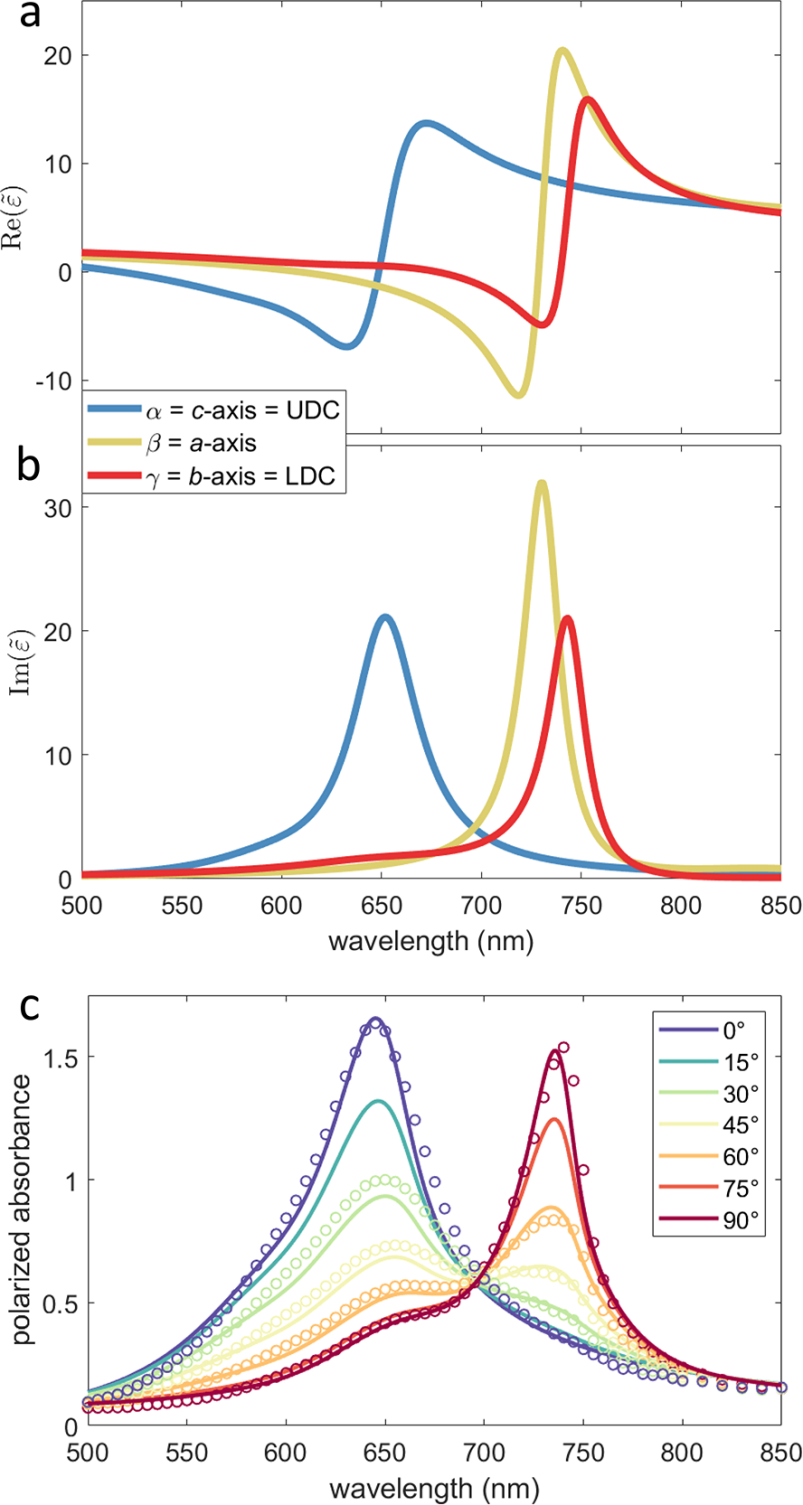
Real (a) and imaginary part (b) of the dielectric tensor components ε_α_ = ε_*c*_ (blue), ε_β_ = ε_*a*_ (yellow), and ε_γ_ = ε_*b*_ (red) are plotted. The UDC is described by ε_*c*_ while the LDC is parallel to γ, thus given by ε_*b*_. In (c) calculated (solid lines) and measured (circles) polarized absorbance spectra of a single SQIB subdomain with (110) alignment and layer thickness *d* = 50 nm are plotted. For 0° the linear polarizer is parallel to the crystallographic *c*-axis and projection of *a*- and *b*-axes for 90°.

The here-determined biaxial dielectric tensor serves well to calculate polarized absorbance spectra (see the Supporting Information for details) for a single SQIB platelet subdomain fully reproducing the measured, projected Davydov splitting (246 meV) including the isosbestic point^17^ (Figure 3c and Figure S12c,d). However, this is large compared to the Davydov splitting of pentacene polymorphs (120–160 meV)^27^ and similar to that of rubrene single crystals (200–300 meV).^22^

All tensor components exhibit extraordinary large values with a strongly negative real permittivity over a small wavelength range just below the excitonic transition. This causes a metal-like shiny-golden appearance of the SQIB platelets to the eye, which is not because of a metal-like behavior (free conduction electrons) but caused by localized Frenkel excitons with large oscillator strength. However, molecular J-aggregated thin films have been demonstrated to support propagating surface exciton–polariton (SEP) modes,^28−30^ just as metallic films support surface plasmon–polariton (SPP) modes. These J-aggregated thin films typically are noncrystalline leading to an effectively isotropic optical response. The crystalline texture of our SQIB samples naturally adds spatial control of the optical response paving the way for nanophotonic functionality.

In conclusion, imaging Mueller matrix ellipsometry is a versatile tool to determine the local dielectric tensor of polycrystalline thin films with unambiguous assignment of tensor components to crystallographic axes. This is highly relevant for a quantitative understanding of microscaled materials in up-to-date optoelectronic applications.

## Supporting Information Available

Detailed description of sample processing, Mueller matrix data fitting routine, Euler angles (eqs S1 and S2), and transfer matrix optical calculation; Figure S1: optical sample images; Figure S2: AFM sample images; Figure S3: polarized spectromicroscopy analysis; Figure S4: knife edge illumination; Figure S5: Theta Scans; Figure S6: Theta Scans parameter correlation; Figure S7: transmission spectroscopic Mueller matrix scans; Figure S8: reflection spectroscopic Mueller matrix scans; Figure S9: spatial Mueller matrix images; Figure S10: oscillator fitting parameters; Figure S11: dielectric tensor for individual ROIs; Figure S12: complex refractive index and calculated absorbance spectra; Table S1: roughness parameters (PDF)
