# Supplementary material for: Determining the Dielectric Tensor of Microtextured Organic Thin Films by Imaging Mueller Matrix Ellipsometry
Source: J Phys Chem Lett. 2021 Mar 19;12(12):3053–8. doi: 10.1021/acs.jpclett.1c00317 (PMC8041376; doi:10.1021/acs.jpclett.1c00317)
Supplement: Supplementary file 1 — jz1c00317_si_001.pdf [file jz1c00317_si_001.pdf]

Electronic Supplementary Information: Determining the Dielectric Tensor of Micro-Textured Organic Thin Films by Imaging Mueller Matrix Ellipsometry

Sebastian Funke,† Matthias Duwe,† Frank Balzer,‡ Peter H. Thiesen,† Kurt Hingerl,¶ and Manuela Schiek*,§,‖

†*Accurion GmbH, Stresemannstr. 30, D-37079 Göttingen, Germany*

‡*Centre for Photonics Engineering, University of Southern Denmark, Alsion 2, DK-6400 Sønderborg, Denmark*

¶*Center for Surface- and Nanoanalytics, Johannes Kepler University, Altenbergerstr. 69, A-4040 Linz, Austria*

§*Institute of Physics, Carl-von-Ossietzky-Str. 9-11, University of Oldenburg, D-26129 Oldenburg, Germany*

‖*present address: Center for Surface- and Nanoanalytics, Johannes Kepler University, Altenbergerstr. 69, A-4040 Linz, Austria*

E-mail: manuela.schiek@jku.at

Sample Preparation and Morphology

2,4-bis[4-(*N,N*-diisobutylamino)-2,6-dihydroxyphenyl]squaraine (SQIB) was synthesized as described before.[1] A 6 mg/mL solution of SQIB in chloroform (Sigma-Aldrich, stabilized with amylene) was spincoated onto (15 mm × 15 mm) float glass substrates (VWR objective slides) with the following settings (SÜSS MicroTec Delta+ 6RC): 1500 rpm (≈50 nm), ramping 5; 3000 rpm (≈50 nm), ramping 3 (≈40 nm); 4000 rpm, ramping 1 (≈30 nm). Approximate layer thicknesses are given in parentheses. This was followed by annealing on a pre-heated hotplate with surface temperature at 180 °C for 2 hours. Polarized microscopy images of the samples are shown in Figure S1. They reveal, that average domain size of the SQIB platelets increase with increasing spin-speed, which correlates with decreasing layer thickness.

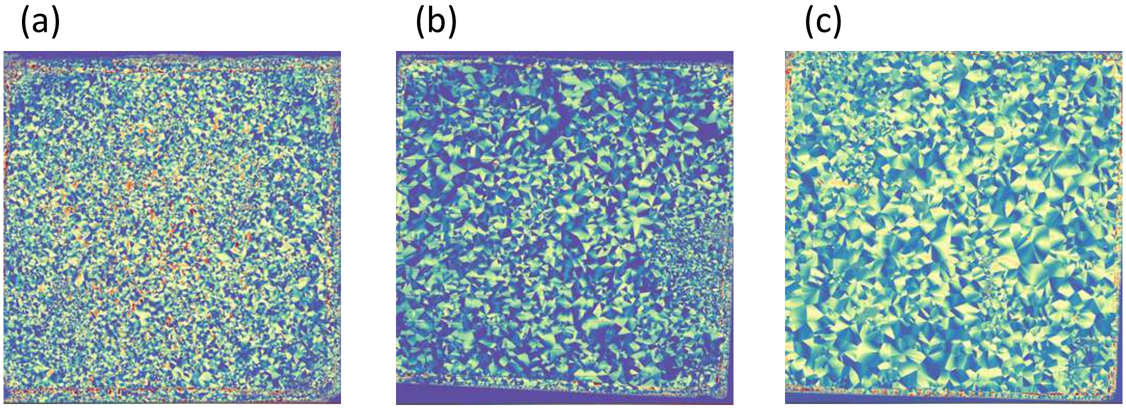

Figure S1: Three samples (size 15 mm × 15 mm) with varying spincoating parameters and subsequent thermal annealing at 180 °C are scanned completely and the images are stitched together. The layer thicknesses of the samples are approximately 50 nm (a), 40 nm (b), and 30 nm (c). Image acquisition parameters: 50° angle of incidence (AOI), LED illumination at 532 nm, PCA-configuration for contrast optimization by eye: P (polarizer) at 60°, C (compensator) at 45°, A (analyzer) at 25°.

Atomic force microscopy images (JPK NanoWizard, intermittent contact mode, Tap300 BudgetSensors cantilever) reveal a certain roughness of the platelets, Figure S2. The wavy texture with a preferred orientation within platelet domains originates from the processing procedure, in particular depend on the crystallization seeds generated by annealing and the following crystallization process. The features become smaller (while domain size increases) with increasing layer thickness / spin-speed. Sample areas have been selected to include

dewetted defect areas, where the underlying glass substrate is visible, to validate the layer thickness deduced from spectroscopic ellipsometry scans. Surface roughness parameters excluding the defect areas are summarized in Table S1. Yet, the surface roughness is small against the probing wavelength and was not included in the ellipsometetric data fitting, see discussion below, Figure S6.

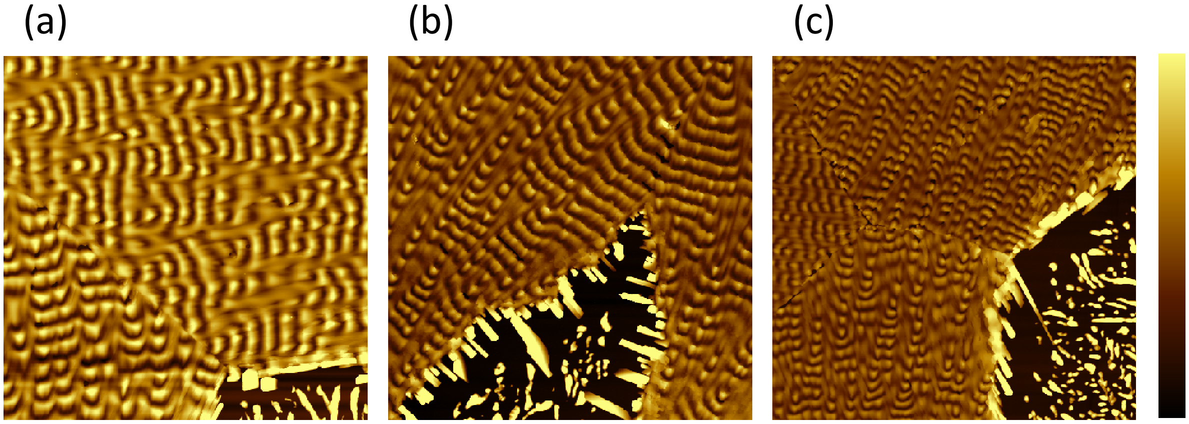

Figure S2: Atomic force microscopy (AFM) images of the three samples shown in Figure S1, (a) thickest sample and slowest spinning, (b) medium thick sample and medium spin speed, (c) thinnest sample and fastest spinning. The AFM images sizes are 40 µm × 40 µm) with an height scale of 125 nm. Surface roughness parameters excluding the defect areas are summarized in Table S1. For image processing Gwyddion has been used.[2]

Table S1: Roughness from AFM Images in Figure S2

sample	thickness (nm)	R_a (nm)	R_q (nm)
(a)	50 ± 5	19 ± 2	23 ± 2
(b)	40 ± 5	14 ± 1	17 ± 1
(c)	30 ± 4	10 ± 1	13 ± 1

[a] Average roughness R_a and root mean squared roughness R_q extracted from AFM images excluding the defect areas in Figure S2 using Gwyddion.

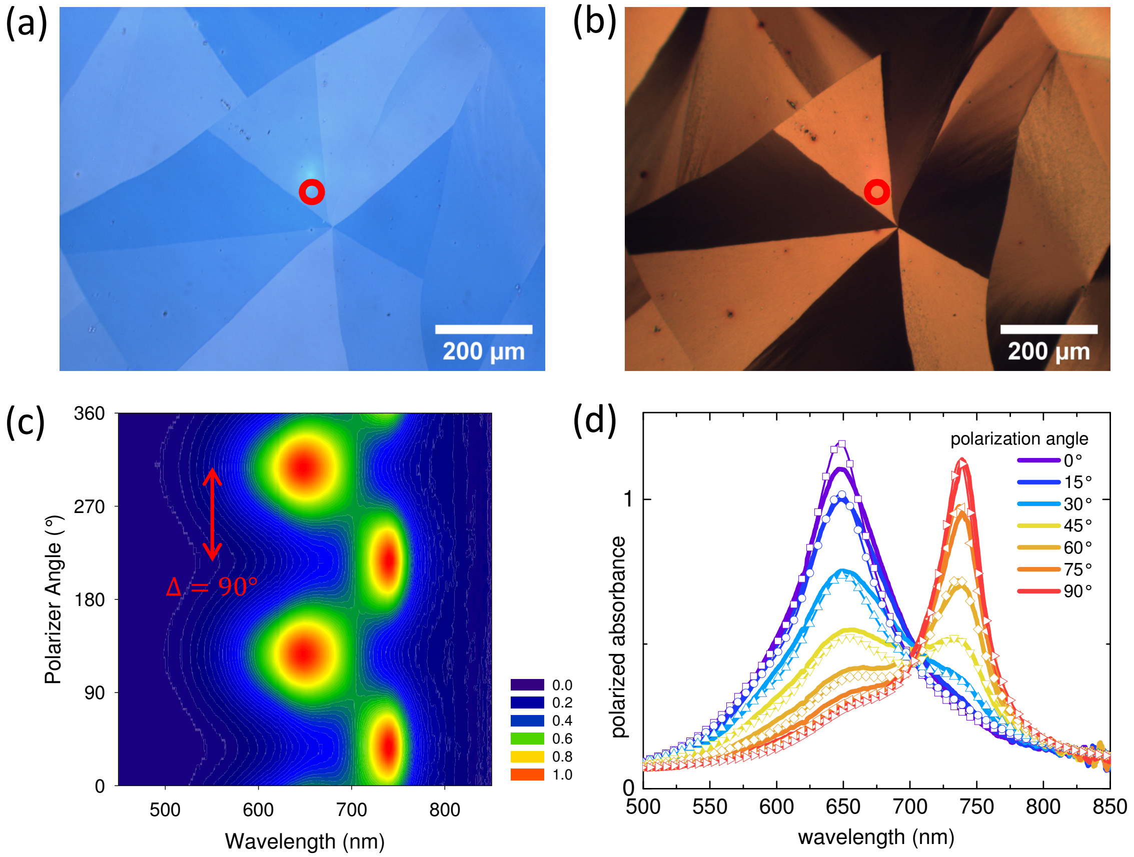

Figure S3: Polarized transmission (single linear polarizer) (a) and reflection (crossed polarizers) microscope images of a thin SQIB sample on glass, annealed at 180 °C, sample (a) in Figures S1 and S2. The red circle marks the position where spectra have been recorded. Polarized absorbance spectra (single linear polarizer), (c) and (d), show the upper (UDC) and lower (LDC) Davydov components.[3] UDC and LDC are polarized mutually perpendicular within the plane, i.e. azimuthal rotation of the linear polarizer from one peak maximum to the other is $\Delta = 90°$ as indicated in (c). The plot in (d) displays both measured (thick solid lines) and calculated (thin lines with markers) polarized absorbance spectra to illustrate their good agreement. For the calculation the complex refractive index as shown in Figure S12 was used, assuming a SQIB layer thickness $d = 32$ nm, following the procedure as outlined in section Transfer Matrix Optical Calculations. Note that all single spectra cross in one point, the so called isosbestic point.[3]

Imaging Mueller Matrix Ellipsometry

Imaging Mueller matrix ellipsometry was performed with a NanoFilm_EP4 system, Accurion GmbH, Göttingen at ambient conditions. It can be operated in principles of nulling and rotating compensator ellipsometry (RCE). The latter is required for recording the Mueller matrix.[4] In addition, ellipsometric contrast imaging allows a visualization and observation of the sample at defined states of polarization of the probing and reflected light. A sketch of the experimental setup is shown in the main paper in Figure 2a. Further details about the setup and the operation principles of the imaging ellipsometer can also be found elsewhere.[4–7]

For the PCSA configuration the collimated beam of monochromatic light is elliptically polarized after passing through the linear polarizer (P) and the compensator (C), which is a rotating quarter waveplate. The elliptically polarized light reflects from (or transmits through) the sample (S) and is collected by a long working distance objective (Nikon, 10×, NA 0.21). After passing the analyzer (A), another linear polarizer, the light is imaged onto a CCD camera. That way, spatially resolution is gained instead of averaging over the light beam spot on the sample. Here, the lateral resolution is 2 µm at a reference wavelength of 400 nm for the 10× objective (Nikon, NA 0.21) used for the measurements.

The applied imaging ellipsometer does not require any focusing of the probing beam to reach its spatial resolution. It actually uses almost collimated light as a probe (NA $\approx$ 0.018, spot diameter $\approx$ 1 mm), and the spatial resolution is purely generated by the microscope imaging in the instrument's detection arm. Due to the low NA of the probing beam, the AOI spread of the ellipsometric measurement could be neglected upon the data analysis. Furthermore, the applied Nikon 10× microscope objective features minimal stress-induced birefringence. The maximum offsets of the measured Mueller matrix elements caused by the objective lens were about ± 0.001 for all applied wavelengths. This is one order of magnitude below the typical deviation observed between the modelled and measured MM-SQIB-spectra and hence did not need to be taken into account upon the model fits.

To obtain the nulling conditions, the positions of P and C are changed that the reflected

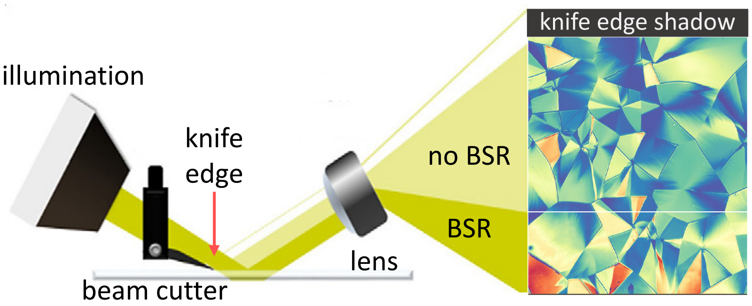

Figure S4: A beam cutter can optionally be moved into part of the illuminating light beam to shade the reflections from the backside of transparent substrates. Since some part of the incoming light is fully blocked by the beam cutter the top part of the image on the CCD camera is black. The middle part is free from backside reflections (BSR), and the lower part is still distorted by backside reflections.

light from the sample is linearly polarized and is extinguished by A so that a minimum signal is detected by the CCD camera. For samples with lateral structure, areas not matching the nulling conditions appear brighter on the detector. In nulling ellipsometry, the so called nulling conditions are used to measure the ellipsometric parameters of a sample. Therefore, the positions of P and C are changed that the reflected light from the sample is linear polarized and is extinguished by a rotation of A to a crossed-polarizer condition. In such way a minimum signal is detected by the CCD-camera. For samples with lateral structure, areas not matching the nulling conditions appear brighter on the detector. The nulling condition depends on thicknesses and refractive indices of the layers investigated. Lateral variations in one or more of these parameters hence may change the observed signal at the CCD camera. This explains the power of the ellipsometric contrast images. P, C, and A are rotatable and offer a high dimensional space of parameters in order to vary the contrast obtained at the CCD camera. It can be either used to visualize small deviations from the known setpoint (e.g. nulling conditions of a certain region) or to maximize the contrast in between certain regions. In the RCE mode, the compensator is rotated and the signal for each pixel is recorded. From the Fourier transform of the signal ellipsometric angles and Mueller matrix values can be evaluated.

The PCSA configuration with rotating compensator allows to record a partial spectroscopic

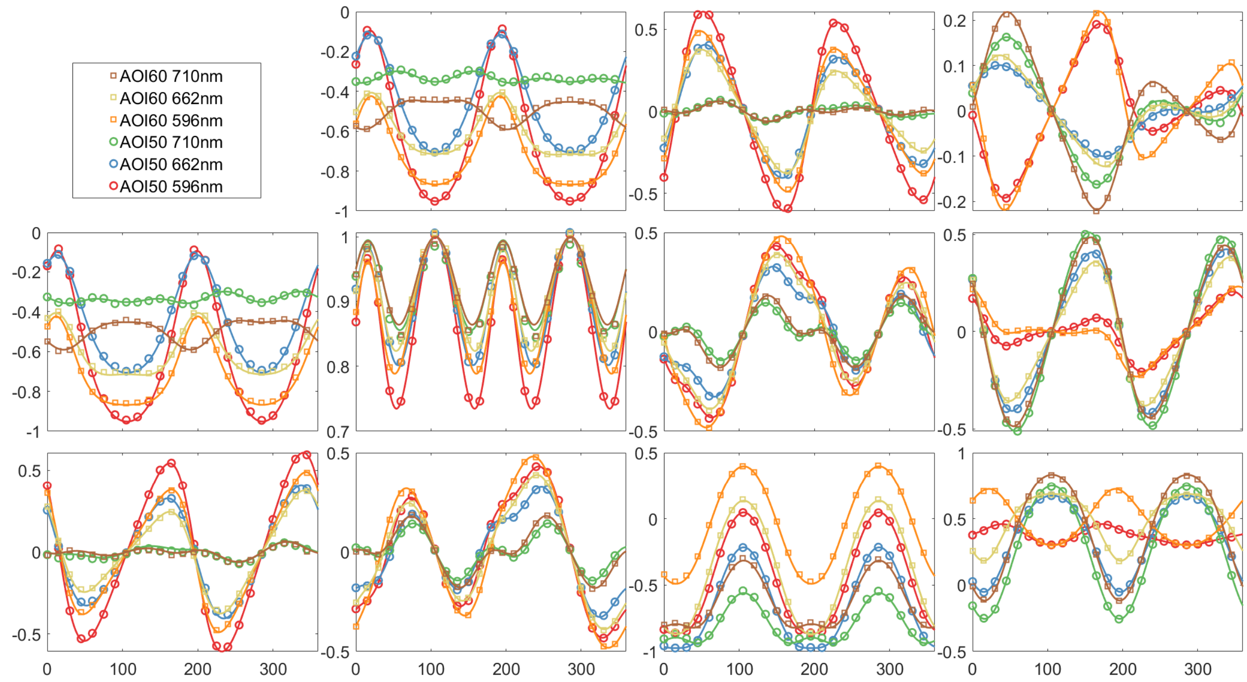

Figure S5: Rotational scans (Theta Scans) in reflection at AOI = 50° (circles) and AOI = 60° (squares) at single wavelengths 596 nm, 662 nm, and 710 nm for ROI 0 (see Figure 2b main paper) are shown. The markers indicate measured data points and the straight lines are fit results. The dots within the plots are measured data points in steps of 15° and the solid lines are fitted data. Y-axes: normalized Mueller matrix values. X-axes: azimuthal rotation angle in degrees.

Mueller matrix, the last row is missing. Since all elements are normalized to $m11$, where $m11$ is set to 1, eleven out of the sixteen elements are recorded. This incomplete Mueller matrix is sufficient for determining the dielectric function of samples with biaxial anisotropy as long as depolarization effects of the light beam are negligible.[8] To avoid depolarization due to incoherent backside reflections from transparent substrates (here glass) knife edge illumination is used. For this a beam cutter is partly moved into the illuminating beam to cause partial shading of the reflections from the backside of the substrate, see Figure S4. Incoherent backside reflections cause blurring of the sample features or ghost images.[7]

As first characterization step, azimuthal rotation scans (Theta Scans)[1] at single wavelengths, here 596 nm (LED, bandwidth 14 nm), 662 nm (LED, bandwidth 22 nm), and 710 nm (mono-

[1]Note that this is azimuthal rotation around the Euler ϕ angle. The phrase "theta" in Theta Scan should not be confused with the Euler θ angle referring to a tilting angle.

chromated Xenon-lamp light, bandwidth 4 nm), have been performed. These scans are recorded in reflection at angles of incidence (AOIs) 50° and 60° in steps of 15° rotational angle. Figure S5 shows exemplarily measured (squares, circles) and fitted (solid lines) data for ROI 0. From such datasets for all ROIs the layer thickness d, the in-plane ϕ orientation of the crystallographic c-axis, and the tilt angle θ of the crystallographic b-axis for the SQIB platelet sub-domains ROI 0 to 13 as indicated in Figure 2b main paper have been determined. The resulting values are summarized in Table 1 main paper. As a figure of merit, the fit returns the root mean square deviation of data points and the optical model: MSE_unbiased $= 1/N \sum_{i=1}^{N} (\mathrm{data_i} - \mathrm{model_i})^2$. For simultaneous fitting of all ROIs: MSE $= 0.0001$.

The angles ϕ, θ and ψ refer to the Euler angles, which denote a rotation of the crystallographic axes with respect to the cartesian laboratory coordinate system x, y, z.[9] Combining the three rotation matrices gives the full transformation matrix $\mathbf{A}$ for the coordinate transformation:

$$\mathbf{A} = \begin{pmatrix} \cos\psi & \sin\psi & 0 \\ -\sin\psi & \cos\psi & 0 \\ 0 & 0 & 1 \end{pmatrix} \begin{pmatrix} 1 & 0 & 0 \\ 0 & \cos\theta & \sin\theta \\ 0 & -\sin\theta & \cos\theta \end{pmatrix} \begin{pmatrix} \cos\phi & \sin\phi & 0 \\ -\sin\phi & \cos\phi & 0 \\ 0 & 0 & 1 \end{pmatrix} \tag{S1}$$

This transformation matrix is applied to the dielectric function tensor $\tilde{\varepsilon}$, which is a second-rank tensor, to give a rotated dielectric tensor $\tilde{\varepsilon}'$ as follows:

$$\tilde{\varepsilon}' = \mathbf{A}\tilde{\varepsilon}\mathbf{A}^{\mathrm{T}} \tag{S2}$$

where $\mathbf{A}^{\mathrm{T}}$ denotes the transpose (inverse) of the transformation matrix $\mathbf{A}$. Coordinate rotation is counter-clockwise. However, due to the definition of the z-axis (Figure 2a main paper) the Euler-ϕ rotation is clockwise in the Mueller matrix images with a starting position at "3 o'clock".

The sample model featured a single, homogenous SQIB layer of unknown thickness, crystal-axis orientation and dielectric tensor elements. The refractive index of the glass substrate was predetermined from a separate reference measurement on an uncoated substrate. The modeling of the surface roughness was omitted to avoid an overly complex model and high correlation of the fit parameters. However, it remains an open question how to include the surface roughness in the optical model properly, as both the typical lateral scales and the amplitudes of the surface features observed in the AFM measurements, Figure S2, are beyond the limits of the effective medium approaches that are typically applied for optical roughness modeling. Interestingly, the simplified model with idealized interfaces yields a very good match to the experimental data. Still, the surface roughness might affect the resulting dielectric tensor elements and the layer thickness values obtained from the fit. Thus, the calculated variations of these quantities for the different examined ROIs might (in parts) be due to the variation of the surface roughness between the different domains.

The set of samples as shown in Figure S1 have been inspected to find a suitable and representative section for the ellipsometric analysis. Although the selection of the 14 ROIs is certainly not unique, it still is a consequence of the applied data analysis approach. First, the Theta Scans required the ROIs to form a circular arrangement close to the image center of rotation, for which obviously the center of the examined platelet was the preferred choice as it allowed for position tracking. If the ROIs had been placed too far off the rotation center, they would partly or completely move out of the field of view for certain sample azimuths upon the Theta Scan. Second, the sample area covered by each ROI needed to be small enough to justify the assumption of a homogeneous SQIB layer (no drift in c-axis orientation) upon modeling (used sizes $140\,\mu m^2$ to $270\,\mu m^2$), but still needed to contain enough pixels to ensure a sufficiently high signal-to-noise ratio (used numbers 400-900 pixels). Finally, the ROIs needed to be placed apart from the domain edges by several microns,

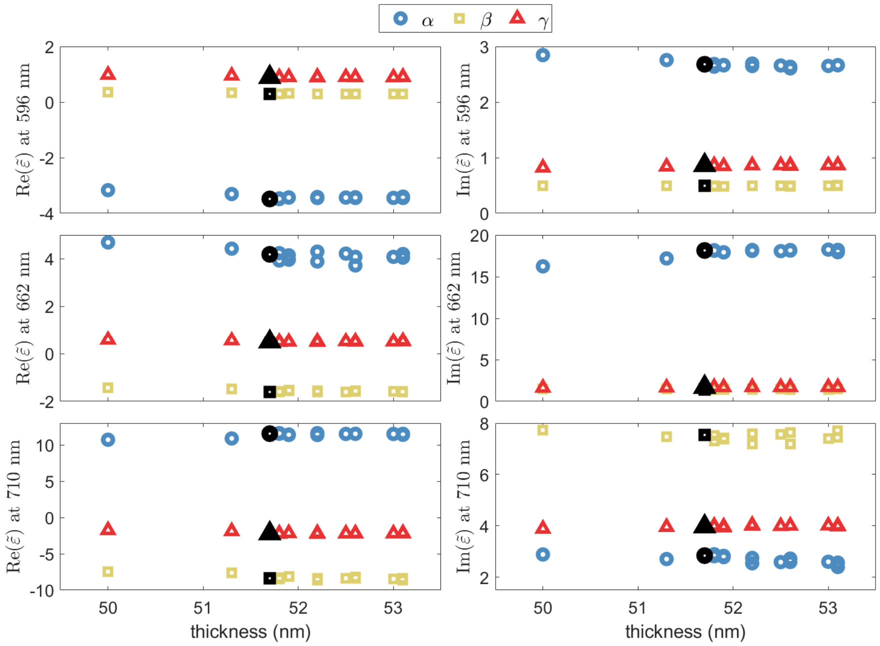

Figure S6: These scatter plots illustrate the variability of the determined layer thickness d for all 14 ROIs as marked in Figure 2b main paper and the associated value of real (Re($\tilde{\varepsilon}$), left column) and imaginary part (Im($\tilde{\varepsilon}$), right column) of the three dielectric tensor components α (blue circles), β (yellow squares), and γ (red triangles); upper row: 596 nm, middle row: 662 nm, lower row: 710 nm. The black markers indicate the results for simultaneous fitting of all ROIs. Y-axes: values of the dielectric tensor components. X-axes: layer thickness d in nanometers.

as otherwise edge effects (diffraction, Airy fringes) might compromise the extracted spectra. A future step of the analysis may be to apply the sample model developed from the ROI-analysis to a pixel-by-pixel conversion of the measured hyper-spectral Mueller-Matrix micrographs. This might allow for a statistically meaningful analysis of the thickness, axis and refractive index distribution across the entire imaged platelet.

To visualize the variability of layer thickness d and its parameter correlation to the values of the dielectric function's tensor elements, they are plotted in Figure S6 as determined from the Theta Scans of all ROIs separately (colored markers) and simultaneously (black markers).

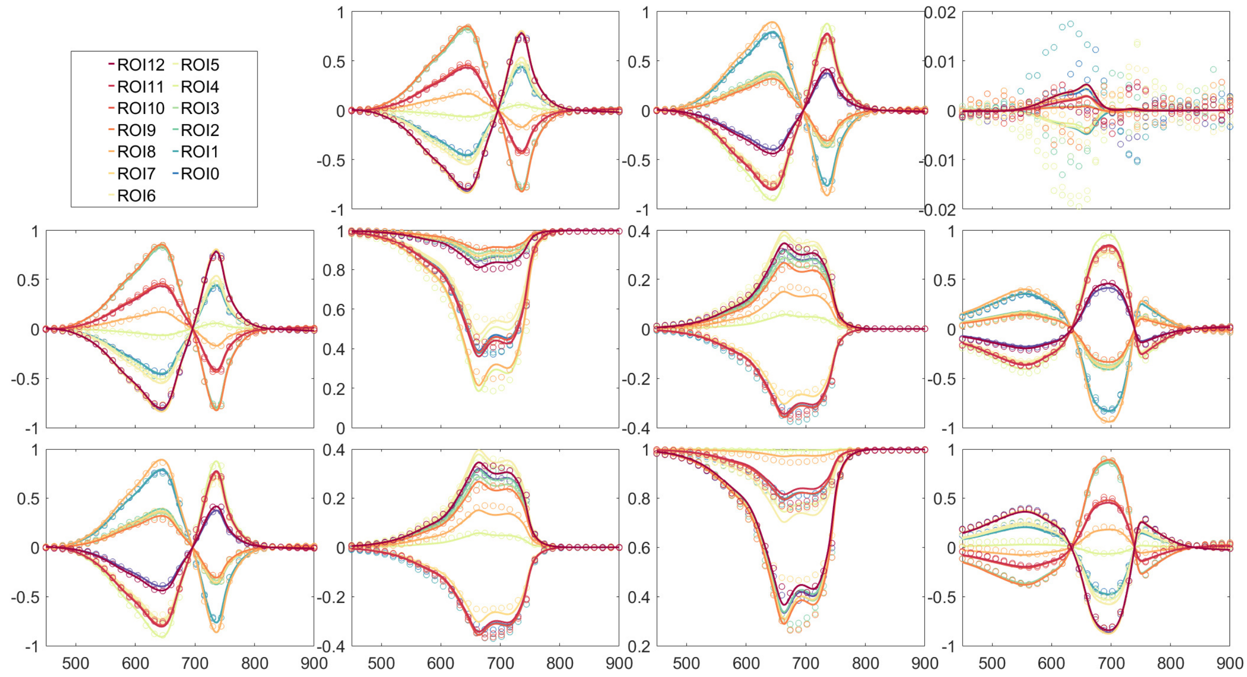

Figure S7: Spectroscopic Mueller matrix scans in normal incidence transmission of 13 subdomains as indicated in Figure 2b main paper. The circles are measured data points (only every 7th data point is shown) and the solid lines are fitted data. Y-axes: normalized Mueller matrix values. X-axes: wavelength in nanometers.

In a second step spectroscopic Mueller matrix data have been recorded with a field of view (FoV) and spatial resolution of (width × height) 785 µm × 1050 µm = 527 × 705 pixels for transmission, and 419 µm × 486 µm = 698 × 811 pixels for reflection under AOI 50°. Each pixel contains information on spatial x-y-position, wavelength and the associated eleven measured Mueller matrix elements. The recordings cover a wavelength range from 450 nm to 900 nm in steps of 2.5 nm for normal incidence transmission, Figure S7, and reflection Mueller matrix measurements, Figure S8. Reflection scans were performed under AOIs of 50° and 60° with the Theta Stage positioned at 0° and 90°.

Interestingly, there appears a significant but poorly fitted signal in the $m14$ element (upper right corner) of the normal incidence transmission scans, Figure S7. These signals are no measurement artifacts and also do not originate from circular dichroism.[10] Instead, they are rather caused by interactions of linear anisotropies due to the polycrystalline nature of the sample and heterogeneity along the light propagation direction.[11]

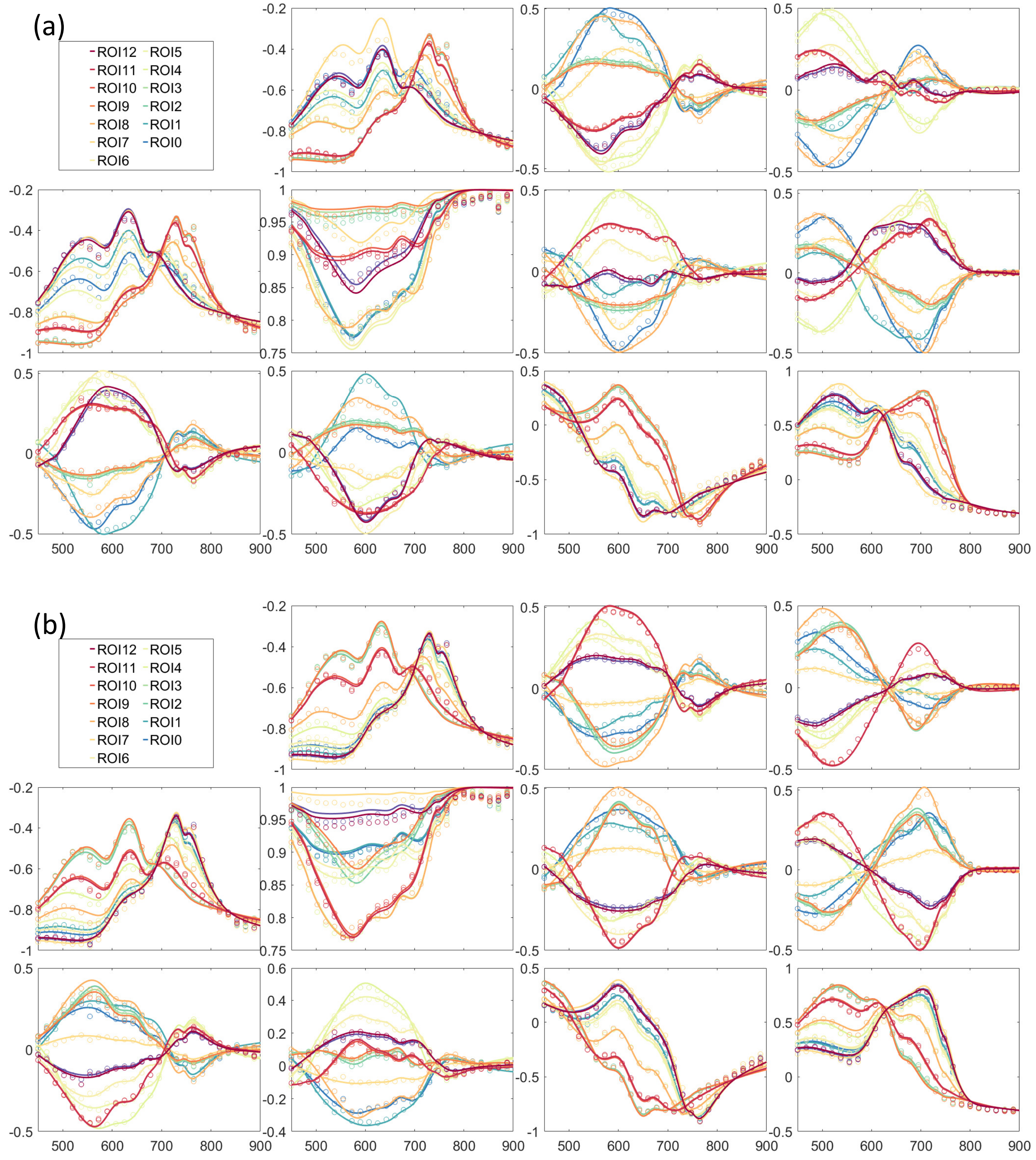

Figure S8: Spectroscopic Mueller matrix scans in reflection AOI 60° of 13 sub-domains as indicated in Figure 2b main paper, (a) Theta Stage at 0° and (b) Theta Stage at 90°. The circles are measured data points (only every 7th data point is shown) and the solid lines are fitted data. Y-axes: normalized Mueller matrix values. X-axes: wavelength in nanometers.

To further visualize the complexity of the acquired data spatial images with microscopic resolution of all 11 Mueller matrix elements at 600 nm wavelength position of the SQIB platelet of choice are displayed in Figure S9 (a) for a transmission and (b) a reflection scan (AOI 50°) and Theta Stage at 0°. Transmission images are cropped to show approximately the same area compared to reflection images. Transmission image sizes (width $\times$ height) 387 µm $\times$ 544 µm = 260 $\times$ 365 pixels. Reflection measurement image sizes (width $\times$ height) 419 µm $\times$ 487 µm = 698 $\times$ 811 pixels.

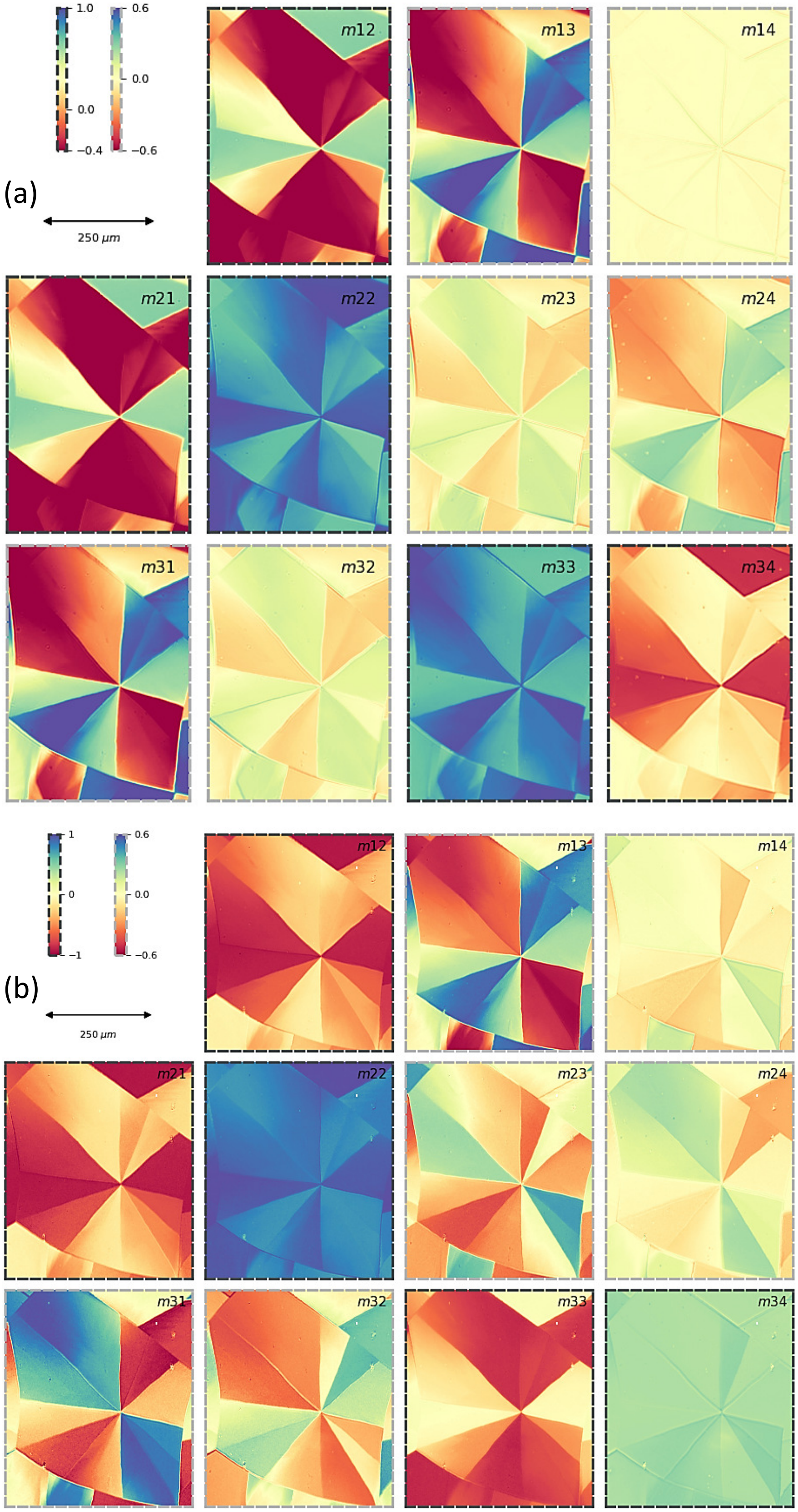

Figure S9: Spatial mapping images of all 11 normalized Mueller matrix elements at 600 nm. (a) Transmission and (b) reflection measurement.

data

		ROI 0	ROI 1	ROI 2	ROI 3	ROI 4	ROI 5	ROI 6	ROI 7	ROI 8	ROI 9	ROI 10	ROI 11	ROI 12	ROI 13	AllData
Alpha	Pole_UV_Ec	6.14506	7.51961	6.31781	7.44794	7.45895	6.00217	5.65955	5.40916	6.15205	7.87275	7.97829	7.52930	8.40261	6.82429	7.05922
	Pole_UV_A	46.55489	78.73082	52.16726	76.69888	77.38961	44.90102	38.16887	36.02269	48.17616	89.42740	90.49532	77.15227	99.58444	60.92835	67.53713
	L1_EC	2.10324	2.09858	2.13019	2.08987	2.09276	2.06502	2.07465	2.10238	2.09363	2.08319	2.08531	2.08971	2.07622	2.09928	2.08689
	L1_A	0.60954	0.79201	0.67479	0.65497	0.59433	0.65966	0.61808	0.99926	0.88057	0.68056	0.60380	0.65504	0.63447	0.78821	0.66447
	L1_tau	0.29988	0.26013	0.28646	0.26788	0.26461	0.25027	0.24998	0.31329	0.27245	0.26196	0.27161	0.27184	0.27044	0.32033	0.27266
	L2_EC	1.90171	1.90163	1.89824	1.90257	1.90229	1.90304	1.90130	1.90388	1.90426	1.90301	1.90226	1.90361	1.90351	1.90136	1.90267
	L2_A	4.64003	4.45120	4.59724	4.51371	4.58963	4.65073	4.70320	4.24043	4.12918	4.50626	4.53617	4.46750	4.51524	4.49337	4.59111
	L2_tau	0.12271	0.10963	0.11103	0.11374	0.11662	0.11434	0.11877	0.12693	0.11925	0.11724	0.11861	0.11400	0.11552	0.11746	0.11658
Beta	Pole_UV_Ec	8.69302	10.52704	12.32410	11.64856	11.80200	15.14509	11.73743	9.08048	11.69149	16.19878	16.63251	17.96492	18.57354	10.85777	11.93354
	Pole_UV_A	104.97655	165.56321	222.71772	196.01930	204.36840	345.74813	207.88211	121.50601	201.97828	401.02545	410.71096	495.72130	534.22987	173.83752	211.97353
	L1_EC	1.48818	1.48067	1.48293	1.46047	1.45810	1.49230	1.48782	1.48415	1.46780	1.46533	1.45677	1.47941	1.46972	1.48600	1.47753
	L1_A	0.21448	0.20027	0.19625	0.36547	0.36792	0.30913	0.29959	0.22414	0.13045	0.30984	0.29965	0.27770	0.27734	0.23456	0.28529
	L1_tau	0.19816	0.19250	0.21243	0.27302	0.28104	0.22662	0.22451	0.25425	0.17852	0.22315	0.23379	0.22109	0.20725	0.21227	0.23214
	TL2_EC	1.69961	1.69259	1.69852	1.69395	1.69161	1.69819	1.69437	1.70001	1.69900	1.69828	1.69604	1.69928	1.69843	1.69914	1.69635
	TL2_A	59.34314	60.81764	55.06659	52.68007	58.77049	62.18295	73.30293	65.63474	51.82093	62.15693	45.63289	56.55544	53.86973	67.97496	59.09599
	TL2_tau	0.05140	0.05285	0.05195	0.05402	0.05040	0.04800	0.04552	0.05472	0.05940	0.05079	0.05939	0.04986	0.04910	0.04867	0.05067
	TL2_Eg	1.42330	1.41430	1.41048	1.40283	1.41415	1.42709	1.44374	1.43657	1.40341	1.42772	1.37999	1.41232	1.40210	1.43927	1.41668
Gamma	Pole_UV_Ec	14.22885	16.16032	12.65046	13.04924	13.26850	9.07426	7.38047	7.28001	9.19893	13.46961	14.57893	15.62774	18.13038	12.63564	14.81928
	Pole_UV_A	336.05054	428.13755	262.33957	266.76075	274.89530	127.74033	81.59469	82.00679	130.82668	295.43791	332.81527	387.75320	529.07830	265.95684	356.44817
	L1_EC	2.53477	2.58751	2.56591	2.57215	2.57163	2.54655	2.53917	2.54885	2.55377	2.58390	2.56691	2.56915	2.56320	2.53147	2.56227
	L1_A	0.01700	0.01947	0.05408	0.01398	0.01289	0.01688	0.03456	0.06080	0.04353	0.00805	0.01115	0.00972	0.00406	0.02681	0.01898
	L1_tau	0.13890	0.13537	0.20529	0.11714	0.11195	0.12874	0.17038	0.20738	0.18180	0.08706	0.11340	0.10740	0.07377	0.14099	0.13764
	L2_EC	1.92314	1.89047	1.89503	1.91901	1.91692	1.92560	1.92127	1.85627	1.85447	1.92049	1.90712	1.92474	1.93687	1.92327	1.91609
	L2_A	0.43355	0.73313	0.73788	0.49003	0.49115	0.49002	0.55966	0.88195	0.75065	0.63231	0.57333	0.50433	0.46168	0.43726	0.53709
	L2_tau	0.29564	0.38883	0.39274	0.30188	0.29795	0.31139	0.32897	0.45284	0.38924	0.33034	0.31830	0.30564	0.28749	0.29501	0.31986
	TL3_EC	1.66368	1.66863	1.66437	1.66780	1.66747	1.66378	1.66493	1.66429	1.66731	1.66616	1.66678	1.66662	1.66592	1.66483	1.66614
	TL3_A	109.58924	74.47527	75.72989	85.49901	88.36010	103.48584	96.25930	73.78880	61.77701	65.78614	88.23033	83.37392	84.06811	101.91941	85.64020
	TL3_tau	0.04844	0.04973	0.04630	0.05088	0.04886	0.04957	0.04999	0.04841	0.05358	0.05265	0.04579	0.05124	0.05144	0.04951	0.05026
	TL3_Eg	1.50099	1.47733	1.47344	1.48563	1.48908	1.49766	1.49330	1.47956	1.46669	1.45691	1.48992	1.48434	1.48161	1.49523	1.48336
	EulerPhiOffsetTrans	-3.53979	-2.653263	-1.92995	-4.261151	-4.107739	-4.583624	-3.990037	-4.129982	-2.72034	-3.320191	-4.084378	-3.393721	-3.505278	-3.33346	-3.629341
	AOIOffset_Trans	-3.294903	2.681012	-2.445309	-1.696126	-1.377547	0.565756	-0.273421	-2.296274	2.117257	0.261497	-0.959802	-3.244135	-2.595214	-2.530894	-1.837544
	root-MSE	0.017028	0.017715	0.018342	0.01876	0.018666	0.017937	0.018026	0.016807	0.018507	0.018535	0.019277	0.017792	0.018179	0.017066	0.018661
	MSE	0.00028995	0.00031382	0.00033643	0.00035194	0.00034842	0.00032174	0.00032494	0.00028248	0.00034251	0.00034355	0.0003716	0.00031656	0.00033048	0.00029125	0.00034823

Pole_UV
Lorentz
Tauc_Lorentz

error

		ROI 0	ROI 1	ROI 2	ROI 3	ROI 4	ROI 5	ROI 6	ROI 7	ROI 8	ROI 9	ROI 10	ROI 11	ROI 12	ROI 13	AllData
Alpha	Pole_UV_Ec	0.00272	0.007015	0.005076	0.006331	0.00593	0.003308	0.002965	0.002824	0.004589	0.005781	0.007034	0.006013	0.007863	0.005273	0.00139
	Pole_UV_A	0.04972	0.165418	0.099296	0.147406	0.139146	0.060456	0.050005	0.048261	0.086266	0.146207	0.17735	0.139133	0.20503	0.109013	0.03046
	L1_EC	0.000051	0.00006	0.000081	0.000057	0.000053	0.000072	0.000073	0.000079	0.000069	0.000045	0.000053	0.000062	0.000056	0.00007	0.00002
	L1_A	0.000314	0.000474	0.000579	0.000403	0.000351	0.000567	0.00054	0.000773	0.000637	0.000339	0.000348	0.000403	0.000376	0.000486	0.00012
	L1_tau	0.000082	0.000099	0.000156	0.000087	0.000082	0.000089	0.000108	0.000112	0.000098	0.000069	0.000078	0.000091	0.000079	0.000098	0.00002
	L2_EC	0.000004	0.000006	0.00001	0.000005	0.000004	0.000007	0.000006	0.000012	0.000009	0.000004	0.000004	0.000005	0.000004	0.000006	0.00000
	L2_A	0.00029	0.00043	0.00047	0.000406	0.000367	0.00055	0.000522	0.000674	0.000587	0.000327	0.000355	0.000389	0.000366	0.000432	0.00011
	L2_tau	0.000012	0.000017	0.000022	0.000015	0.000014	0.000019	0.000018	0.000031	0.000027	0.000012	0.000013	0.000014	0.000012	0.000017	0.00000
Beta	Pole_UV_Ec	0.009582	0.028861	0.0441	0.037207	0.03625	0.094192	0.042408	0.016687	0.040269	0.099606	0.099203	0.130061	0.137097	0.025541	0.00956
	Pole_UV_A	0.25552	0.968204	1.669482	1.322062	1.324687	4.443597	1.585822	0.487438	1.463565	5.066764	5.030054	7.349121	8.063342	0.869892	0.35773
	L1_EC	0.00006	0.000081	0.00009	0.000074	0.000082	0.000074	0.000079	0.000124	0.000093	0.000052	0.000065	0.000066	0.000056	0.000075	0.00002
	L1_A	0.000234	0.000348	0.000311	0.000478	0.000494	0.00042	0.000433	0.00042	0.000277	0.000333	0.000384	0.000337	0.000299	0.000311	0.00010
	L1_tau	0.000216	0.000298	0.00031	0.00034	0.000374	0.000282	0.000293	0.000436	0.000353	0.000227	0.000294	0.000248	0.000223	0.000271	0.00008
	TL2_EC	0.000013	0.000017	0.000017	0.00002	0.000019	0.00002	0.000019	0.000017	0.000017	0.000016	0.000018	0.000018	0.000018	0.000016	0.00000
	TL2_A	0.037914	0.050258	0.050585	0.043191	0.045591	0.061765	0.072739	0.060302	0.044361	0.045052	0.030826	0.048307	0.043777	0.057958	0.01260
	TL2_tau	0.00002	0.000026	0.000027	0.000029	0.000027	0.00003	0.000029	0.00003	0.000029	0.000023	0.000025	0.000027	0.000025	0.000026	0.00001
	TL2_Eg	0.000094	0.000122	0.000137	0.000132	0.00012	0.000145	0.000134	0.000129	0.000132	0.000107	0.000119	0.00013	0.000128	0.000118	0.00003
Gamma	Pole_UV_Ec	0.038691	0.070142	0.04669	0.036185	0.035698	0.013993	0.0074	0.007249	0.014631	0.031513	0.044897	0.060126	0.089891	0.035854	0.01409
	Pole_UV_A	1.884824	3.809007	2.006219	1.53711	1.535531	0.427866	0.184702	0.184305	0.44763	1.435468	2.113301	3.064593	5.351829	1.568793	0.69760
	L1_EC	0.000138	0.000132	0.000185	0.000142	0.000129	0.000168	0.000154	0.000131	0.000134	0.000104	0.000139	0.000166	0.000212	0.000127	0.00004
	L1_A	0.000079	0.000089	0.000215	0.000072	0.000062	0.0001	0.000152	0.000194	0.000144	0.000038	0.000057	0.000063	0.00004	0.00011	0.00002
	L1_tau	0.000592	0.000553	0.000743	0.000576	0.00052	0.000703	0.000655	0.000549	0.00054	0.000406	0.00056	0.000668	0.000776	0.000529	0.00016
	L2_EC	0.000039	0.000056	0.000071	0.000042	0.000038	0.00005	0.000051	0.000084	0.000074	0.000031	0.000037	0.000041	0.000035	0.000049	0.00001
	L2_A	0.000347	0.000446	0.000635	0.000391	0.000351	0.000488	0.000541	0.000547	0.000487	0.000326	0.00036	0.000384	0.000308	0.000432	0.00012
	L2_tau	0.000152	0.000144	0.000196	0.000156	0.000139	0.000205	0.000197	0.000169	0.000145	0.000112	0.000126	0.00016	0.000141	0.000189	0.00004
	TL3_EC	0.000008	0.000009	0.00001	0.000011	0.000011	0.000011	0.000011	0.00001	0.00001	0.000008	0.000009	0.000009	0.000009	0.00001	0.00000
	TL3_A	0.063682	0.055296	0.091677	0.058915	0.053953	0.084215	0.089948	0.088412	0.057875	0.039567	0.054515	0.056034	0.048485	0.078148	0.01689
	TL3_tau	0.000011	0.000014	0.000019	0.000014	0.000013	0.000015	0.000015	0.000021	0.00002	0.00001	0.000012	0.000013	0.000012	0.000014	0.00000
	TL3_Eg	0.000047	0.000071	0.000113	0.000062	0.000054	0.000067	0.000079	0.000113	0.000096	0.000062	0.000055	0.00006	0.000053	0.000064	0.00002
	EulerPhiOffsetTrans	0.00085	0.001444	0.00254	0.000997	0.001002	0.001025	0.000952	0.002152	0.002295	0.000793	0.000726	0.00094	0.000619	0.001404	0.000224
	AOIOffset_Trans	0.003912	0.006719	0.011159	0.009365	0.008238	0.006572	0.0058	0.011113	0.009855	0.005766	0.007444	0.005348	0.004438	0.006044	0.001253

Figure S10: Upper panel: Fit parameter values for individual and combined fitting of all ROIs. Lower panel: Errors for the fit parameter values listed above. The errors refer to the numerical fitting by the Levenberg-Marquardt routine and do not consider, e.g., the bandwidth of the monochromator, which limits the experimental energy resolution to 0.01 eV-0.03 eV for the applied spectral range. Abbreviations: L = Lorentz oscillator. TL = Tauc-Lorentz Oscillator. EC = center energy in eV. A = amplitude. tau = broadening in eV. Eg = energy gap in eV.

As a first step of the determination of the spectroscopic SQIB dielectric tensor elements from the Mueller matrix data, cubic-spline functions were employed to obtain a best match of the single-layer model to the experimental data without enforcing Kramers-Kronig consistency. The latter means that ε_1 and ε_2 were fitted independently for each of the three tensor elements. Supporting points, however, were equidistant and the same for the corresponding sets of ε_1 and ε_2. The layer thickness d, tilt angle θ and the ϕ orientation were used as determined from the Theta Scans for the following fitting procedure of the spectroscopic Mueller matrix data. However, a ϕ-offset was allowed for the transmission data to account for a different sample adjusting. Reflection and transmission data were fitted simultaneously but individually for each ROI. Then, to obtain Kramers-Kronig consistency of the dielectric tensor components, the fit was parameterized with sets of Lorentz and Tauc-Lorentz oscillators as listed in Figure S10.

The parameterization was conducted with a self-programmed routine in Python using the NumPy[12] and SciPy[13] packages. The routine was programmed to interact with the software EP4_Model from Accurion to calculate the Mueller matrix values from a given set of layer parameters (thickness of SQIB layer, dielectric tensors of SQIB, air and glass, Euler angles of SQIB layer). The Python routine was programmed to process in a 2-fold manner with an intermediate step. For all steps the experimental spectral data of all 14 ROIs are used (transmission and reflection Mueller matrix values). Thickness and Euler angles are taken from the Theta Scan fit. In this 2-fold manner approach each routine is done first for each ROI separately, and finally for all ROIs simultaneously.

At first, a spline fitting routine was programmed. Therefore, the spectral dielectric tensor of the SQIB is described by a cubic-spline evaluation of supporting points. It shall be noted, that for the α, β and γ axes a separate spline evaluation is undertaken. The 40 supporting points (nodes) are placed equidistantly in the wavelength domain from 450 nm to 900 nm. Equidistantly spaced wavelength nodes were chosen to have sufficient nodes for the description of the observed peaks in the dielectric tensor. The fit is done using the 'TRF'

option of the SciPy Curve_fit function. The comparison of the modeled and experimental data is done in the Python environment, however the calculation of the modeled Mueller matrix values is done via EP4_Model. During each iteration of the fit the spline functions are evaluated and changed by the fitting algorithm in order to obtain the best fitting of experimental and modeled Mueller matrix values. The routine is used to implement the spline fitting, automation and simultaneous fitting of transmission and reflection Mueller matrix values, that is not usable from the user interface of the EP4_Model. This way, a spline description of the dielectric tensor is obtained. The agreement of experimental and modeled values is good, but has no direct physical meaning and utilizes more than 240 (40 nodes for n and k and each axes) fit parameters during a single fitting run.

To minimize the number of fit parameters a parametric modeling is introduced. Lorentz and Tauc-Lorentz functions are implemented and used to describe the dielectric function of each axis of the SQIB layer. It allows to calculate the dielectric response of a set of Lorentz and Tauc-Lorentz terms. In the intermediate step the Curve_fit function is used to find optimal start parameters for the Lorentz and Tauc-Lorentz terms by fitting the parametric functions to the result of the spline approach for each axis. For the final fitting via Python the parametric approach, instead of the spline approach, is used to calculate the dielectric tensor during the fitting. The results denote values for center energies, widths and strengths of the oscillators. For Tauc-Lorentz terms the band gap is an additional parameter obtained. The final results can be seen in Figure S10. The intermediate results are not shown here. This 2-fold approach by first evaluating the dielectric tensor via a spline interpolation to have starting point for the parametric approach in combination with the simultaneous fitting of transmission and reflection Mueller matrix data obtained at different AOIs and Theta angles, helped to de-correlate dependencies in thickness and dielectric tensor.

The uncertainty values of the fit results ("error" table in Figure S10) were obtained from the fit-parameter covariance matrix that is provided by the Levenberg-Marquardt (LM) fit routine. It should be noted that these values are estimates based on the assumption that only

the dependent variables (i.e. the measured MM data) are subject to a measurement uncertainty (standard deviation). In contrast, the LM routine does not consider the uncertainty values of the independent measurement parameters, most notably the probing wavelength and the AOI. Hence, the uncertainty values provided in S10 do not consider e.g. the intrinsic bandwidth of the monochromator (about 0.01 eV-0.03 eV for the applied spectral range). Thus, the LM-estimated uncertainty values of e.g. an oscillator resonance energy may be much smaller (about 10^{-4} eV) than the monochromator's resolution. The total precision or uncertainty of the measurement of any resonance energy, of course, cannot be better than the energy resolution of the applied instrumentation.

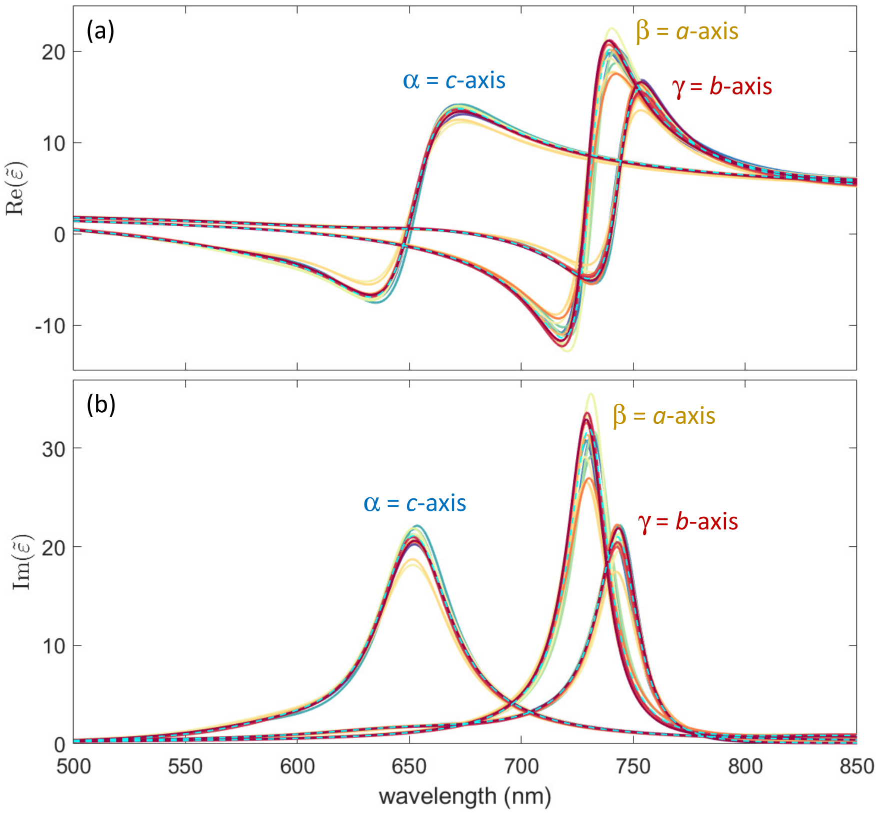

Figure S11: All tensor components of the dielectric function for independent fitting of all 14 ROIs. Reflection and transmission data for each ROI are fitted simultaneously. Dashed cyan lines are the result for combined fitting as shown in the main text in Figure 3. (a) Real part and (b) imaginary part.

The final best fit result for jointly fitting of all ROIs for the dielectric tensor is displayed in Figure S11 together with the individual fits for each ROI. In Figure S12 the data are shown in the representation as complex refractive index N, real refractive index n in (a) and extinction coefficient k in (b). Here, the vibronic progression as typical for the UDC[14] is clearly visible as a shoulder in the extinction coefficient, blue curve Figure S12 (b).

The peak position of the measured / calculated absorbance spectra, Figure S12 (c) and Figure 3(c) main paper, are at 645 nm (1.922 eV) for UDC and 740 nm (1.676 eV) for LDC resulting in a Davydov splitting energy of 246 meV. Remember that these values are for the projected UDC and LDC onto the (1 1 0) crystal face.

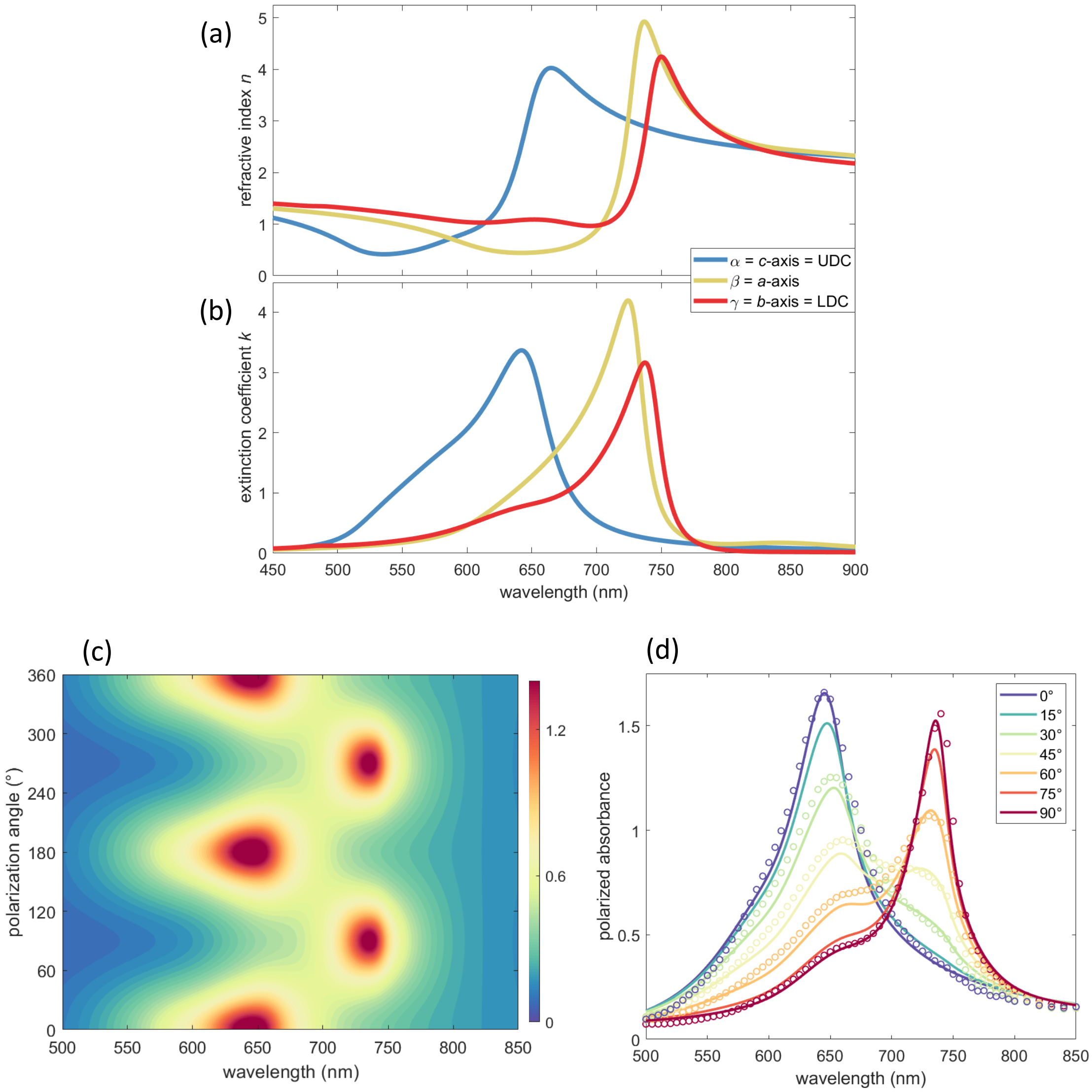

Figure S12: Complex refractive index of the SQIB tensor components (a) refractive index n and (b) extinction coefficient k. From these the polarized absorbance for a single SQIB domain with $d = 50\,\text{nm}$ and $(1\,1\,0)$ orientation is calculated via a Transfer Matrix approach and shown as contour plot in (c). These are the same calculated data as in the main manuscript in Figure 3c. For polarizer position at 0° the polarization is parallel to the crystallographic c-axis (UDC, tensor component α), and for polarizer position 90° the polarization is parallel to the projection of the crystallographic a- and b-axes. In (d) measured (circles) and calculated (solid lines) polarized absorbance spectra with a second linear polarizer parallel to the first one used as analyzer ("pp"-transmission) in the beam path, i.e. $-\log T_{\text{pp}}$. Note that in this case no isosbestic point (single crossing point of all spectra) is visible.

Transfer Matrix Optical Calculations

Polarized absorbance spectra can be calculated via a Transfer Matrix approach. For this, N_α (c-axis, UDC) was used together with an effective refractive index N_{eff} for the projected a- and b-axes to account for the (1 1 0) alignment of the SQIB platelet. The effective refractive index N_{eff} is calculated as follows:[15]

$$N_{\text{eff}} = \frac{N_\beta \cdot N_\gamma}{\sqrt{\sin\theta^2 \cdot N_\beta^2 + \cos\theta^2 \cdot N_\gamma^2}} \tag{S3}$$

with θ=50° to account for the tilt angle of the b-axis. N_α and N_{eff} are mutually perpendicular within the (1 1 0) plane. The incoming light is linear polarized and chosen to be parallel for the starting angle 0° to the c-axis (N_α, UDC). Upon azimuthal rotation by 90° the polarization is then parallel to the projected a- and b-axis (N_{eff}). As transparent substrate a thick, incoherent layer with a refractive index determined earlier for the float glass substrate is implemented via absolute squares of Fresnel coefficients to simulate the 1 mm thick glass substrate.[16]

Calculated polarized absorbance spectra can be seen in Figures S12 (c) and (d).

Note that in our previous studies[3,17] the assignment of UDC and LDC to the crystallographic axes was interchanged due to the ambivalence in interpreting simple polarized absorbance spectra under normal incidence. The unique assignment of dielectric tensor components to the crystallographic axes was only possible by imaging Mueller matrix ellipsometry.

References

(1) Abdullaeva, O. S.; Schulz, M.; Balzer, F.; Parisi, J.; Lützen, A.; Dedek, K.; Schiek, M. Photoelectrical Stimulation of Neuronal Cells by an Organic Semiconductor-Electrolyte Interface. *Langmuir* **2016**, *32*, 8533–8542.

(2) Nečas, D.; Klapetek, P. Gwyddion: An Open-Source Software for SPM Data Analysis. *Open Physics 10*, 181–188.

(3) Balzer, F.; Kollmann, H.; Schulz, M.; Schnakenburg, G.; Lützen, A.; Schmidtmann, M.; Lienau, C.; Silies, M.; Schiek, M. Spotlight on Excitonic Coupling in Polymorphic and Textured Anilino Squaraine Thin Films. *Cryst. Growth Des.* **2017**, *17*, 6455–6466.

(4) Duwe, M.; Quast, J.-H.; Schneider, S.; Fischer, D.; Beck, U. Thin-Film Metrology of Tilted and Curved Surfaces by Imaging Mueller-Matrix Ellipsometry. *J. Vac. Sci. Technol. B* **2019**, *37*, 062908.

(5) Braeuninger-Weimer, P.; Funke, S.; Wang, R.; Thiesen, P.; Tasche, D.; Viöl, W.; Hofmann, S. Fast, Noncontact, Wafer-Scale, Atomic Layer Resolved Imaging of Two-Dimensional Materials by Ellipsometric Contrast Micrography. *12*, 8555–8563.

(6) Funke, S.; Wurstbauer, U.; Miller, B.; Matković, A.; Green, A.; Diebold, A.; Röling, C.; Thiesen, P. Spectroscopic Imaging Ellipsometry for Automated Search of Flakes of Mono- and n-Layers of 2D-Materials. *421*, 435–439.

(7) Funke, S.; Miller, B.; Parzinger, E.; Thiesen, P.; Holleitner, A.; Wurstbauer, U. Imaging Spectroscopic Ellipsometry of MoS_2. *J. Phys.: Condens. Matter* **2016**, *28*, 385301.

(8) Beaudry, N. A.; Zhao, Y.; Chipman, R. Dielectric Tensor Measurement from a Single Mueller Matrix Image. *JOSA A* **2007**, *24*, 814–824.

(9) Fujiwara, H. *Spectroscopic Ellipsometry: Principles and Applications*; John Wiley & Sons, 2007.

(10) Schulz, M.; Zablocki, J.; Abdullaeva, O. S.; Brück, S.; Balzer, F.; Lützen, A.; Arteaga, O.; Schiek, M. Giant Intrinsic Circular Dichroism of Prolinol-Derived Squaraine Thin Films. *Nat. Commun.* **2018**, *9*, 2413.

(11) Freudenthal, J. H.; Hollis, E.; Kahr, B. Imaging Chiroptical Artifacts. *Chirality* **2009**, *21*, E20–E27.

(12) Harris, C. R.; Millman, K. J.; van der Walt, S. J.; Gommers, R.; Virtanen, P.; Cournapeau, D.; Wieser, E.; Taylor, J.; Berg, S.; Smith, N. J. et al. Array Programming with NumPy. *Nature* **2020**, *585*, 357–362.

(13) Virtanen, P.; ; Gommers, R.; Oliphant, T. E.; Haberland, M.; Reddy, T.; Cournapeau, D.; Burovski, E.; Peterson, P.; Weckesser, W. et al. SciPy 1.0: Fundamental Algorithms for Scientific Computing in Python. *Nat. Methods* **2020**, *17*, 261–272.

(14) Hestand, N. J.; Spano, F. C. Expanded Theory of H-and J-Molecular Aggregates: The Effects of Vibronic Coupling and Intermolecular Charge Transfer. *Chem. Rev.* **2018**, *118*, 7069–7163.

(15) Wahlstrom, E. *Optical Crystallography*; Wiley: New York, 1969.

(16) Katsidis, C. C.; Siapkas, D. I. General Transfer-Matrix Method for Optical Multilayer Systems with Coherent, Partially Coherent, and Incoherent Interference. *Appl. Opt.* **2002**, *41*, 3978–3987.

(17) Balzer, F.; Abdullaeva, O. S.; Maderitsch, A.; Schulz, M.; Lützen, A.; Schiek, M. Nanoscale Polarization-Resolved Surface Photovoltage of a Pleochroic Squaraine Thin Film. *Phys. Status Solidi B* **2020**, *257*, 1900570.
